# Basophil-specific deletion of interleukin 15 impairs memory CD8^+^ T cell homeostasis in the bone marrow

**DOI:** 10.3389/fimmu.2026.1842739

**Published:** 2026-08-18

**Authors:** Carmen Stecher, Ines Neuwirth, Amra Begunic, Nina Braun, Dietmar Herndler-Brandstetter

**Affiliations:** Center for Cancer Research, Comprehensive Cancer Center Vienna, Medical University of Vienna, Vienna, Austria

**Keywords:** basophil, bone marrow, interleukin 15, memory CD8 T cell, tissue-resident CD8 T cell

## Abstract

Basophils develop in the bone marrow and are associated with allergic inflammation but also contribute to protective immune responses against pathogens. Upon activation, they produce large amounts of cytokines, including interleukin (IL)-4 and IL-13. However, their physiological functions during homeostasis remain poorly defined. Here, using single-cell RNA sequencing and cytokine knock-in reporter mice, we identify basophils as the immune cell population with the highest IL-15 expression among immature and mature bone marrow leukocytes. Conditional deletion of IL-15 in basophils using *Mcpt8-Cre* mice results in a decrease of central-memory and CD69^+^ tissue-resident memory CD8^+^ T cells during steady-state conditions. In contrast, the homeostatic maintenance of other IL-15-dependent immune cell lineages, such as NK and NKT cells, was unaffected. Together, these findings identify basophils as a key source of IL-15 in the bone marrow that selectively supports memory CD8^+^ T cell maintenance.

## 1 Introduction

The bone marrow provides specialized niches supporting the long-term maintenance and self-renewal of hematopoietic stem cells (HSCs) as well as hematopoiesis. The maintenance of HSCs is regulated by endothelial cells and perivascular mesenchymal progenitor cells that produce C-X-C motif chemokine ligand 12 (CXCL12) and stem cell factor (1). In addition, HSC niches can produce distinct lineage-instructive signals, including interleukin (IL)-7, to regulate multipotent progenitor differentiation (2). However, the bone marrow also serves as a major reservoir for immunological memory, including plasma cells, memory B cells and memory CD4^+^ T cells (3, 4). In addition, the bone marrow is a major reservoir and site of recruitment for central-memory CD8^+^ T cells (5) as well as a priming site for T cell responses to blood-borne antigen (6). Bone marrow niches secrete distinct cytokines and promote cell-cell interactions that collectively regulate the maintenance and homeostatic proliferation of memory immune cells. For example, stromal and myeloid cells produce survival factors such as APRIL, BAFF and IL-6 that promote the long-term persistence of plasma cells in the bone marrow (3). Noteworthy, basophils and eosinophils also significantly contribute to the survival of plasma cells in the bone marrow by secreting IL-6 and APRIL (7, 8).

In addition, the bone marrow constitutes an IL-15-rich microenvironment that supports the development and survival of IL-15-dependent immune cell lineages. Studies using IL-15 reporter mice have demonstrated that stromal and hematopoietic cells are a significant source of IL-15 in the bone marrow (9–11). Conditional knockout studies in mice have shown that IL-15 expression in macrophages and dendritic cells regulates the survival of memory CD8^+^ T cells and NK cells in the bone marrow (12, 13). In addition, distinct IL-15-expressing stromal cells contribute to the maintenance of memory CD8^+^ T cells, NK cells and NKT cells in the bone marrow (11, 14). And the human bone marrow has also been shown to host polyfunctional memory CD4^+^ and CD8^+^ T cells with close contact to IL-15-producing cells (15). Although IL-15 expression has been reported in a variety of hematopoietic cell types, including monocytes, dendritic cells, granulocytes and hematopoietic stem and progenitor cells, the functional relevance of IL-15 from these populations has so far only been investigated for monocytes and dendritic cells using conditional knockout mouse models (10). Despite evidence that basophils participate in cytokine-driven crosstalk with T cells and other immune cells, the role of basophil-derived IL-15 in immune cell regulation under physiological conditions has not yet been investigated.

Basophils are rare granulocytes derived from bone marrow hematopoietic progenitors that circulate as terminally differentiated cells with limited proliferative capacity. Basophils are potent effector cells of the innate immune system, primarily implicated in inflammatory responses to allergens, but they also contribute to host defense against pathogens (16). Beyond their classical association with type 2 inflammation, they are now recognized as multifunctional regulators of immunity with context-dependent roles (17). Basophils can act as antigen-presenting cells in allergen-induced T helper type 2 responses and enhance immunological memory by producing key cytokines such as IL-4 and IL-6, particularly in the bone marrow and spleen upon antigen restimulation (18). These cytokines drive B cell activation and improve the capacity of CD4^+^ T cells to provide B cell help, underscoring the important contribution of basophils to humoral memory immune responses (19). Additionally, basophils function as critical communicators within the immune system, including priming of group 2 innate lymphoid cells (ILC2s) to respond to neuron-derived signals that support tissue integrity (20). Recently, basophils have also been implicated as contributors to the development of renal fibrosis (21).

In this study, we use single-cell transcriptomics and flow cytometric analysis of IL-15 knock-in reporter mice, and identify basophils as the cell population with the highest IL-15 expression among immature and mature bone marrow leukocytes. Conditional deletion of IL-15 in basophils leads to a decrease in central-memory and CD69^+^ tissue-resident memory CD8^+^ T cells during steady-state conditions. Our results therefore demonstrate that basophils are an important source of IL-15 in the bone marrow and selectively support memory CD8^+^ T cell homeostasis in the bone marrow.

## 2 Materials and methods

### 2.1 Experimental animals

Mice were housed at the Core Facility Laboratory Animal Breeding and Husbandry (Medical University of Vienna) in individually ventilated cages under controlled environmental conditions. Animal experiments were performed in accordance with institutional and national guidelines and approved by the Austrian Federal Ministry of Education, Science and Research (GZ 66.009/0407-V/3b/2018 and GZ 66.009/0408-V/3b/2018).

IL-15-IRES-EGFP knock-in mice were described previously (11). The IL-15-IRES-EGFP mouse model generates a bicistronic mRNA, and EGFP positivity demonstrates that the mRNA is present, has reached the cytoplasm and is being translated, at least the IRES-dependent EGFP portion. The strength of the EGFP signal does not necessarily correlate with the amount of IL-15 protein being produced or secreted, since post-transcriptional regulation of IL-15 protein synthesis has been reported. Mast cell protease 8 (Mcpt8)-Cre: YFP (Basoph8) mice (22) were purchased from the Jackson Laboratory (JAX #017578). IL-15^fl^ mice were generated by Nan-Shih Liao (23) (Academia Sinica, Taiwan; MTA #13T-1050130-16M) and obtained from the Jackson Laboratory (JAX #034188).

All Cre-loxP-based conditional knockout experiments were conducted using littermate controls matched for age and sex. Mcpt8-Cre: YFP mice were backcrossed to IL-15^fl^ mice lacking any Cre-positive ancestry to minimize the risk of propagating unintended recombination. Mice annotated as *Mcpt8-Cre* in this manuscript were all heterozygous for Mcpt8-Cre: YFP (*Mcpt8^Cre/wt^*). All mice were screened for germline recombination using deletion-specific genotyping primers.

For the short-term depletion of basophils, IL-15-IRES-EGFP mice were injected i.p. with PBS or anti-mouse FcϵR1α (MAR-1) antibody (5 µg in 100 µL, Biolegend, MAR-1, purified, Cat# 134302) (24) once per day on days 1–3 and 7-9. The depletion efficacy of basophils in the bone marrow of control and MAR-1-treated mice was assessed at day 10.

### 2.2 Genotyping

Genomic DNA for animal genotyping was isolated from toe or ear biopsies by proteinase K digestion followed by heat inactivation. PCR amplification was performed using standard conditions, and products were resolved on 2% agarose gels for genotype determination. The following primers were used: Il15 floxed (F: AGAGCCTTGCCACTAGCTACA; R: AGAAGGCTTTGCAATGTTCA), Il15 deleted (F: AGGATGCAGGAGATGTTTGG; R: CCTCTGCACCTTGACTGGTT), Mcpt8-Cre (WT F: GCTCTTCCACCTCCTCAGTG; Cre F: CCAGCCATCTGTTGTTTGC; common R: GGGATGAGGATGGTTGCTTA), and Il15-GFP (WT F: GCCACTTGGATCACATGAAA; GFP F: ACTTCAAGATCCGCCACAAC; common R: CACACACAGCCAAACACACA).

### 2.3 Bioinformatic analysis of public datasets

#### 2.3.1 scRNA-seq

Publicly available murine bone marrow single-cell RNA sequencing (scRNA-seq) datasets were re-analyzed using R (version 4.5.2) and Seurat (version 5.4.0). Datasets from the Tabula Muris consortium (droplet marrow dataset) and the bone marrow dataset published by Baccin et al. (25) were downloaded from the Tabula Muris consortium (26) data portal and the Gene Expression Omnibus (GEO; accession GSE122465, total bone marrow sample GSM3466897), respectively. Raw count matrices were imported into Seurat with a minimum threshold of 3 cells per gene and 200 detected genes per cell. Data were normalized and scaled, and highly variable genes were identified using Seurat’s FindVariableFeatures function (selection method “vst” with nfeatures = 2000). To enable joint analysis and correct for batch effects, datasets were integrated using the Harmony R package. Dimensionality reduction was performed by principal component analysis (PCA, npcs = 15) followed by visualization using uniform manifold approximation and projection (UMAP) based on the first 15 Harmony components. Cell clusters were identified using a graph-based clustering approach implemented in Seurat (FindNeighbors/FindClusters) at a resolution of 0.7. Cluster-defining genes were identified using Seurat’s differential expression framework, and expression patterns were visualized using heatmaps generated with the ComplexHeatmap R package, where color intensity represents scaled average expression and dot size indicates the fraction of expressing cells. IL-15-expressing cells were defined as cells with detectable Il15 transcripts (unique molecular identifier (UMI) count >0). The proportion of Il15-positive cells and average Il15 expression were calculated per cluster using Seurat’s FetchData and AverageExpression functions, respectively.

#### 2.3.2 Xenium spatial transcriptomics analysis

Spatial transcriptomics data were obtained from the publicly available Mouse Bone Xenium dataset (8% formic acid decalcification) generated by 10x Genomics (27) and analyzed using R (version 4.5.2) and Seurat (version 5.4.0). Raw Xenium output files were imported using ReadXenium, and gene expression matrices were used to generate a Seurat object via CreateSeuratObject. Cells with zero detected transcripts were excluded, and an additional filtering step was applied to only retain cells with more than 5 total transcript counts (nCount_Xenium > 5). Data were normalized using variance-stabilizing transformation, followed by scaling and dimensionality reduction using principal component analysis (PCA; npcs = 30). Cells were then embedded using uniform manifold approximation and projection (UMAP) based on the first 30 principal components. Clustering was performed using a graph-based approach (FindNeighbors and FindClusters at a resolution of 0.3). Cluster marker genes were identified using the FindAllMarkers function, and the top 10 differentially expressed genes for each cluster were selected based on average log2 fold-change. Gene expression patterns across clusters were visualized by a heatmap using the ComplexHeatmap package. Cell type annotation was performed manually based on cluster-specific marker gene expression, resulting in identification of major hematopoietic and stromal populations, including myeloid, lymphoid, erythroid, endothelial, stromal, and basophil-enriched clusters. Low-quality clusters were identified and annotated separately. For visualization, clusters were displayed using DimPlot and spatially mapped using the ImageDimPlot function. Spatial expression of selected genes (e.g., *Mcpt8*) was visualized using ImageFeaturePlot and molecule overlay plots. For region-specific analyses, fields of view were cropped using spatial coordinate ranges, and selected areas were re-visualized to highlight local cellular organization and gene expression patterns.

Spatial relationships between cell types were analyzed using the CRAWDAD (Cell-type Relationship Analysis Workflow Done Across Distances) R package (28). CRAWDAD quantifies colocalization or spatial separation between cell types across multiple spatial length scales using permutation-based null models. Briefly, neighborhoods were defined around cells of a reference cell type, and spatial trends were evaluated across increasing distance scales. Neighborhood distance was determined to be optimal at 15µm. Cell-type labels were permuted within spatial tiles to generate a null distribution, enabling calculation of z-scores representing enrichment or depletion of neighboring cell types relative to a random expectation. Statistical significance thresholds were corrected for multiple testing using the Bonferroni method. To summarize pairwise spatial relationships, CRAWDAD dot plots were generated using the vizColocDotplot function, where the dot color represents the strength and direction of spatial association as a z-score, and the dot size represents the spatial scale at which the relationship first becomes statistically significant. Positive z-scores indicate spatial colocalization, whereas negative z-scores indicate spatial avoidance. For visualization of spatial cluster distribution, vizClusters was used with a neighborhood distance of 15 µm.

### 2.4 Tissue preparation

Bone marrow cells were isolated from femurs and tibiae of both hind limbs as performed previously (29). Briefly, bones were dissected and freed of surrounding muscle and connective tissue using sterile gauze and forceps, and epiphyses were removed. Bone marrow cavities were flushed with cold FACS buffer (PBS supplemented with 2% fetal bovine serum (FBS)) using a 25G needle and syringe. Splenocyte suspensions were prepared by gentle mechanical dissociation of spleens through a 70 μm cell strainer using the plunger end of a syringe, followed by rinsing with FACS buffer. Peripheral blood was collected by terminal retro-orbital bleeding from isoflurane-anesthetized mice into heparin-coated tubes to prevent coagulation. For all tissues, red blood cells were lysed using ACK lysis buffer (150mM NH_4_Cl, 10mM KHCO_3_, 0.1mM Na_2_EDTA, pH 7.4)) for 2–5 minutes at room temperature. Blood samples underwent two consecutive rounds of ACK lysis. After washing with 2 mL FACS buffer, cells were counted, filtered through a cell strainer and processed for downstream flow cytometry staining.

### 2.5 Quantitative RT-PCR

RNA was extracted from sorted cells (sorted directly into RLT Plus lysis buffer with 40mM DTT) using the RNeasy Micro Plus kit (Qiagen; Cat# 74034) according to the manufacturer’s instructions. cDNA was synthesized with the RevertAid First Strand cDNA synthesis kit (Thermo Fisher Scientific; Cat# K1621), using the supplied oligo(dT)18 primers. Quantitative reverse transcription PCR (qRT-PCR) was performed on a CFX96 Touch Real-Time PCR Detection System (BioRad) using the Maxima SYBR Green master mix including ROX reference dye (Thermo Fisher Scientific; Cat# K0222). Sequence-specific oligonucleotide primers were synthesized by Sigma Aldrich. Relative expression values were normalized to mouse β-actin using the comparative threshold cycle method (2^−ΔCt^, or 2^−ΔΔCt^ for fold changes between samples). The following primers were used for qRT-PCR (11, 14): β-actin (forward: GCTCTTTTCCAGCCTTCCTT, reverse: CTTCTGCATCCTGTCAGCAA), Il15 (forward: GTGACTTTCATCCCAGTTGC, reverse: GCAAGGTAGAGCACGTTTC), Il15ra (forward: GACACCAAAGGTGACCTCACAG, reverse: CTGTCTCTGTGGTCATTGCGGT), Il3ra (forward: CTGGCATCCCACTCTTCAGAT, reverse: GGTCCCAGCTCAGTGTGTA), Il4 (forward: CCCCAGCTAGTTGTCATCCTG, reverse: CAAGTGATTTTTGTCGCATCCG), Mcl1 (forward: GACGACCTATACCGCCAGTC, reverse: AGAGGCTTCGAGTCCTTGGA) and Mki67 (forward: ATCATTGACCGCTCCTTTAGGT, reverse: GCTCGCCTTGATGGTTCCT).

### 2.6 Flow cytometry

Single-cell suspensions were blocked for at least five minutes using a CD16/32 blocker (Mouse Fc block TruStain FcX PLUS, Biolegend, Cat# 156604) and then stained in a volume of 100-200 μL FACS buffer with commercial fluorochrome-conjugated antibodies for 40–60 minutes on ice. The following antibodies were used (all from Biolegend unless otherwise indicated):

CD115 (AFS98, BV605, Cat# 135517), CD117 (2B8, BV785, Cat# 105841), CD11b (M1/70, PerCP, Cat# 101230), CD127 (A7R34, PE, Cat# 135009), CD200R3 (Ba13, PE, Cat# 142205), CD3ϵ (500A2, APC-Fire750, Cat# 152308; 500A2, APC, Cat# 152306; 145-2C11, BV421, Cat# 100336), CD4 (RM4-5, AF700, Cat# 100536), CD44 (IM7, BV785, Cat# 103059), CD45 (30-F11, AF700, Cat# 103128; APC-Fire750, Cat# 103154), CD49a (HM1a, PE-Cy7, Cat# 142608), CD49b (DX5, APC-Cy7, Cat# 108920; APC, Cat# 108910), CD62L (MEL-14, PE-Cy7, Cat# 104417), CD69 (H1.2F3, PE, Cat# 104507), CD8α (53-6.7, BV650, Cat# 100742; BB700, BD Biosciences, Cat# 566410), CXCR3 (CXCR3-173, BV421, Cat# 126522), FcϵR1α (MAR-1, APC, Cat# 134315; biotin, Cat# 134303), IFN-γ (XMG1.2, APC, Cat# 505810), Ly6C (HK1.4, PE-Cy7, Cat# 128017), Ly6G (1A8, APC-Fire750, Cat# 127651), NK1.1 (PK136, BV421, Cat# 108732), T-bet (4B10, AF488, Cat# 644830), TCRβ (H57-597, APC-Fire750, Cat# 109246), and Ter119 (Ter119, BV421, Cat# 116233; APC-Cy7, Cat# 116223). Biotinylated antibodies were detected using fluorochrome-conjugated streptavidin (BV785, Biolegend, Cat# 405249; BV421, BD Biosciences, Cat# 563259). Dead cells were excluded using the Zombie Aqua Viability stain (Biolegend, Cat# 423101) or Ghost Dye Violet 510 (Cytek Biosciences, Cat# SKU 13-0870-T100).

For intracellular cytokine staining, cell suspensions from bone marrow and spleen were suspended in RPMI-1640 medium (in-house recipe) supplemented with 10% FBS and stimulated with Cell Activation Cocktail (PMA/Ionomycin mixture, Cat# 423301, Biolegend) and Brefeldin A (Cat# 420601, Biolegend) according to the manufacturer’s instructions for 4 hours at 37 °C and 5% CO_2_. An unstimulated control without Cell Activation Cocktail was run on the same plate for each organ. After stimulation, cell suspensions were washed with FACS buffer and prepared for surface antibody staining. Following the surface marker staining step, cells were fixed for 15 minutes using a paraformaldehyde-based fixation buffer (Biolegend, Cat# 420801) and permeabilized according to the manufacturer’s instructions. Intracellular antibodies were stained overnight at 4 °C. Samples were acquired on a BD LSR Fortessa X-20 (BD Biosciences). Compensation and data analysis were performed using FlowJo version 10 (BD Biosciences). Gating strategy included exclusion of debris (FSC-A vs SSC-A), doublets (FSC-A vs FSC-H), and dead cells (viability dye-positive). Detailed gating strategies are provided in the **Supplementary Figures**.

### 2.7 Statistical analysis

Sample processing was performed in a randomized order, and experimental groups were defined based on genotype. Data are presented as mean ± standard error of the mean (SEM) from biological replicates. Statistical analyses were conducted using GraphPad Prism (version 8). Comparisons between two groups were performed using unpaired two-tailed Student’s *t* tests. For comparisons involving three or more groups, one-way analysis of variance (ANOVA) followed by Tukey’s multiple comparisons test was applied. A *P* value of < 0.05 was considered statistically significant. *P < 0.05, **P < 0.01, ***P < 0.001, ****P < 0.0001 and ns, not significant *P* > 0.05. Where applicable, *P* values above 0.05 are reported as numbers in the respective graphs.

## 3 Results

### 3.1 Single-cell transcriptomics identifies basophils as a prominent IL-15-expressing population in the bone marrow

**Figure 1:**
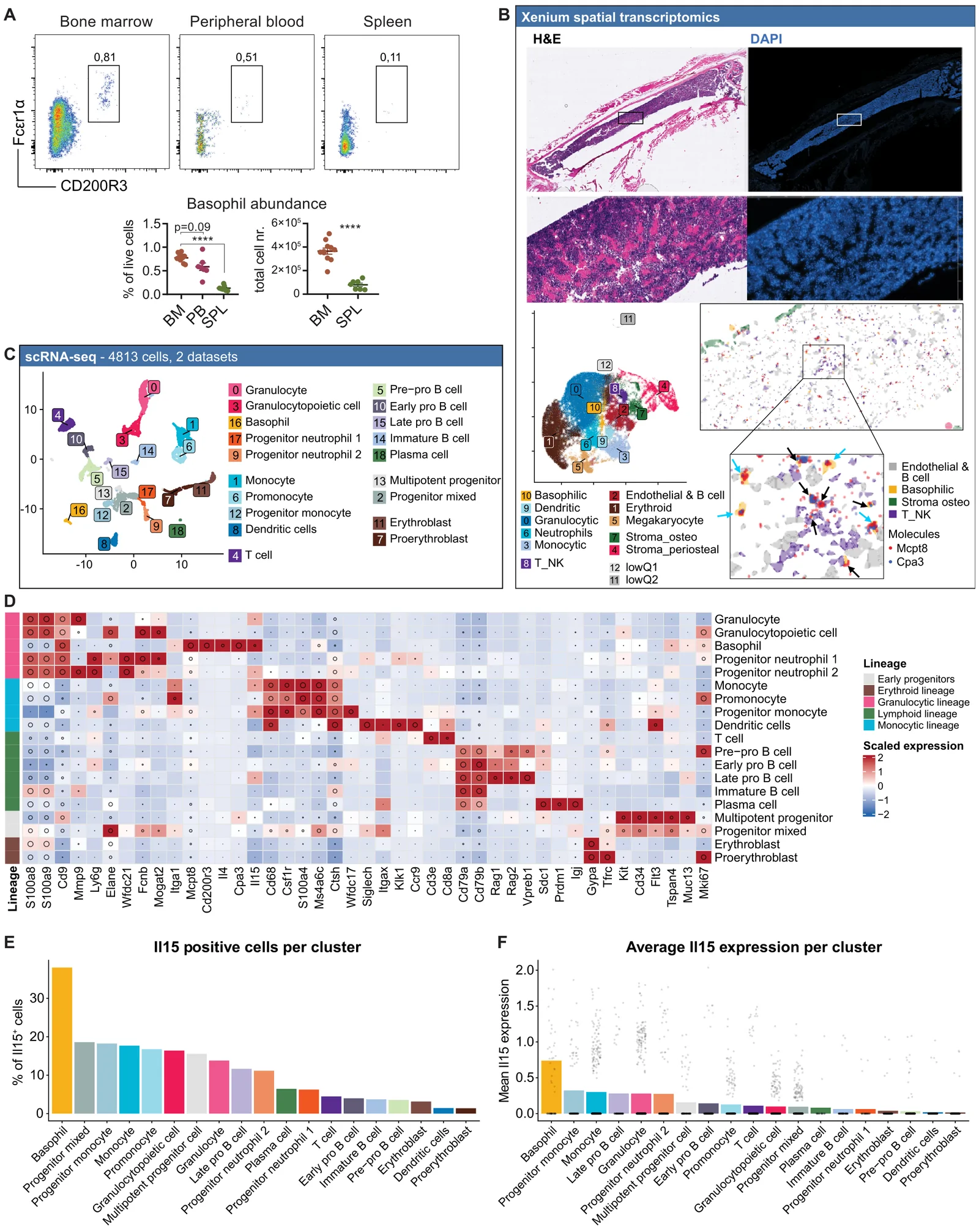
Single-cell transcriptomics identifies basophils as a prominent IL-15-expressing population in the bone marrow. **(A)** Representative flow cytometry plots (top) and quantification (bottom) of basophils (FcϵR1α^+^CD200R3^+^) in murine bone marrow, peripheral blood, and spleen. Cells were pre-gated on live, single cells negative for a lineage cocktail (Ter119, CD3ϵ and CD20) and lacking Ly6G and CD117 expression. Dot plots show mean ± SEM. Asterisks and numbers indicate *P* values (****P < 0.0001) compared to bone marrow calculated using one-way ANOVA with Dunnett’s *post hoc* test or Student’s two-tailed unpaired *t* test, respectively. **(B)** Spatial transcriptomics analysis of a murine femur using a publicly available Xenium dataset (27). Top: hematoxylin and eosin (H&E) and DAPI staining with a highlighted region used for detailed spatial visualization. Bottom: dimensionality reduction plot showing all identified cell clusters and ImageDimPlot of the enlarged region highlighting endothelial, basophilic, and stromal osteogenic cell populations alongside expression of the basophil markers *Mcpt8* and *Cpa3*. Black arrows indicate basophils in contact with T_NK and cyan arrows indicate basophils not in contact with T_NK. **(C)** Dimensionality reduction plot showing harmony-integrated scRNA-seq datasets of murine bone marrow cells derived from two independent studies (25, 26) with a total of 4,813 cells. Clusters are annotated and visually grouped according to their hematopoietic lineage. **(D)** Heatmap showing scaled expression of curated lineage-defining marker genes across clusters in the integrated scRNA-seq dataset. Circles represent the fraction of cells that express the gene. **(E)** Bar graphs showing the percentage of *Il15*-expressing cells per cluster in the integrated scRNA-seq dataset. **(F)** Bar graphs showing mean *Il15* expression per cluster in the integrated scRNA-seq dataset.

Basophils are emerging as multifunctional, context-dependent regulators of immunity and have been shown to support plasma cell survival in the bone marrow. Interestingly, a previous study suggests that basophils may express IL-15 under steady-state conditions (30). However, the role of basophil-derived IL-15 in immune cell regulation under physiological conditions has not yet been investigated. We therefore first assessed the abundance of basophils across hematopoietic tissues. Flow cytometric analysis revealed that FcϵR1α^+^CD200R3^+^ basophils are considerably more abundant in the murine bone marrow than in peripheral compartments such as blood and spleen, both in relative frequency and absolute numbers (**Figure 1A**). We therefore investigated whether basophils might contribute to IL-15 production within the steady-state bone marrow microenvironment. To first characterize the spatial distribution of basophils, we re-analyzed a publicly available Xenium spatial transcriptomics dataset of the murine femur (27). Basophilic cells, identified by expression of *Mcpt8* and *Cpa3*, were distributed throughout the bone marrow cavity without clear enrichment in endosteal or perivascular regions (**Figure 1B**; **Supplementary Figures 1A–C**). Of note, about 60% of basophils, highlighted by black arrows, were in contact with CD3^+^CD8^+^ T cells (T_NK; **Figure 1B**). To further characterize spatial relationships between basophils and other bone marrow cell types, we performed spatial cell-type relationship analysis using CRAWDAD (28). This analysis revealed that basophils displayed a negative spatial association with other basophils, consistent with a dispersed distribution rather than local clustering within the marrow microenvironment (**Supplementary Figures 1D, E**). Similarly, CD3^+^CD8^+^ T cells (T_NK) showed a negative spatial association with other CD8^+^ T cells, when using CRAWDAD analysis. Next, we analyzed IL-15 expression in basophils relative to other hematopoietic populations by integrating two publicly available murine bone marrow scRNA-seq datasets (25, 26). After harmony-based integration, the combined dataset comprised 4,813 cells and included clusters representing multiple hematopoietic lineages and differentiation stages (**Figure 1C**). Cluster identities were confirmed using curated lineage-defining marker genes (**Figure 1D**). Within this integrated dataset, the basophil cluster (defined by expression of *Mcpt8*, *Cd200r3*, *Il4*, and *Cpa3*) surprisingly contained the highest proportion of IL-15-expressing cells (**Figure 1E**) and exhibited the highest average *Il15* transcript levels among all annotated clusters (**Figure 1F**). Other hematopoietic populations, including typical IL-15 producers such as monocytes, also expressed detectable levels of *Il15*, but at substantially lower frequencies and expression levels. Together, these data identify basophils as a prominent source of IL-15 within the steady-state bone marrow, suggesting that basophils may contribute to regulatory signaling within the bone marrow microenvironment beyond their established roles in peripheral immune responses.

### 3.2 IL-15 reporter mice identify basophils as the highest IL-15-expressing myeloid subset in the bone marrow

**Figure 2:**
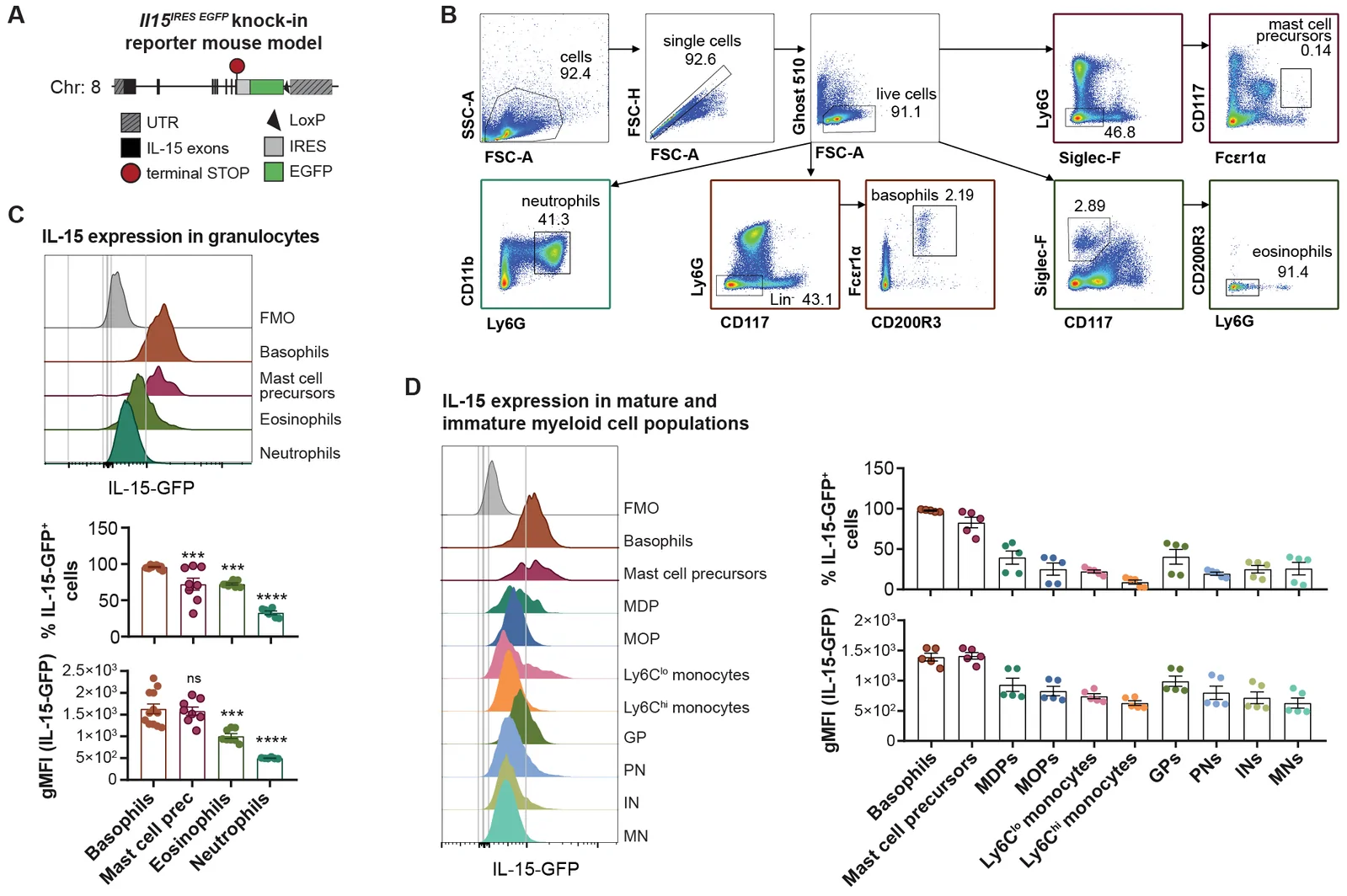
IL-15 reporter mice identify basophils as the highest IL-15-expressing myeloid subset in the bone marrow. **(A)** Schematic representation of the IL-15-GFP knock-in reporter mouse model. An IRES-EGFP cassette was inserted downstream of the endogenous *Il15* stop codon, resulting in bicistronic expression of IL-15 and EGFP from the native *Il15* locus (11). **(B)** Flow cytometric gating strategy used to identify basophils, mast cell precursors, eosinophils, and neutrophils in the bone marrow of IL-15-GFP mice. **(C)** Representative histograms (top) and quantification (bottom) of IL-15-GFP expression in granulocyte populations of the bone marrow. Bar graphs show the percentage of IL-15-GFP^+^ cells and the geometric mean fluorescence intensity (gMFI) of GFP for the indicated cell types. **(D)** IL-15-GFP expression across mature and immature myeloid cell populations in the bone marrow. Representative histograms (left) and quantification of the percentage of IL-15-GFP^+^ cells and gMFI of GFP (right) are shown for basophils, mast cell precursors, monocyte-dendritic progenitors (MDPs), monocyte progenitors (MOPs), Ly6C^hi^ and Ly6C^lo^ monocytes, granulocyte precursors (GPs), pre-neutrophils (PNs), immature neutrophils (INs) and mature neutrophils (MNs). All dot plots show mean ± SEM. Asterisks indicate significant differences to bone marrow from one-way ANOVA and Tukey’s multiple comparison test. ***P < 0.001; ****P < 0.0001; ns, not significant.

Our single-cell transcriptomic analyses suggested that basophils represent a prominent source of IL-15 in the bone marrow. However, IL-15 expression is challenging to quantify accurately at the mRNA level due to its low transcript abundance and technical limitations inherent to scRNA-seq, which can lead to underestimation of its expression (11, 31). Furthermore, detection of IL-15 protein by conventional antibody-based approaches remains technically challenging. Thus, to directly assess IL-15 expression in hematopoietic cell populations, we employed our previously described IL-15 knock-in reporter mouse model (11) in which an IRES-EGFP cassette was inserted downstream of the endogenous Il15 stop codon, resulting in bicistronic expression of IL-15 and EGFP from the native Il15 locus to faithfully reflect endogenous IL-15 transcription (**Figure 2A**).

Using this reporter model, we first analyzed IL-15-GFP expression across granulocyte populations in the bone marrow by flow cytometry. Basophils, mast cell precursors, eosinophils, and neutrophils were identified using established surface marker combinations (**Figure 2B**). Strikingly, basophils displayed the highest frequency of IL-15-expressing cells, with virtually all basophils exhibiting detectable IL-15-GFP expression. In addition, basophils showed the highest reporter signal intensity among the granulocyte populations analyzed, indicating particularly robust IL-15 expression. In contrast, eosinophils and neutrophils contained significantly lower proportions of IL-15-GFP positive cells and displayed reduced reporter intensity (**Figure 2C**).

Given the cellular complexity of the bone marrow and the presence of numerous myeloid differentiation stages, we next investigated IL-15 expression across a broader spectrum of immature and mature myeloid cell populations. These included monocyte-dendritic progenitors (MDPs), monocyte progenitors (MOPs), Ly6C^hi^ and Ly6C^lo^ monocytes, granulocyte precursors (GP), pre-neutrophils (PN), immature neutrophils (IN) and mature neutrophils (MN), alongside basophils and mast cell precursors (**Supplementary Figure 2**). Consistent with our initial observations, basophils again exhibited the highest frequency of IL-15-GFP positive cells and the strongest reporter expression among the analyzed populations (**Figure 2D**). Although IL-15 baseline expression was detectable in certain monocytic populations, including Ly6C^hi^ and Ly6C^lo^ monocytes, both the proportion of IL-15-GFP-expressing cells and reporter intensity remained markedly lower than in basophils.

Together, these data confirm that basophils represent an IL-15-producing population within the steady-state bone marrow and suggest that they may contribute substantially to IL-15 availability in the bone marrow microenvironment.

### 3.3 Basophil-derived IL-15 is dispensable for NK cell homeostasis

**Figure 3:**
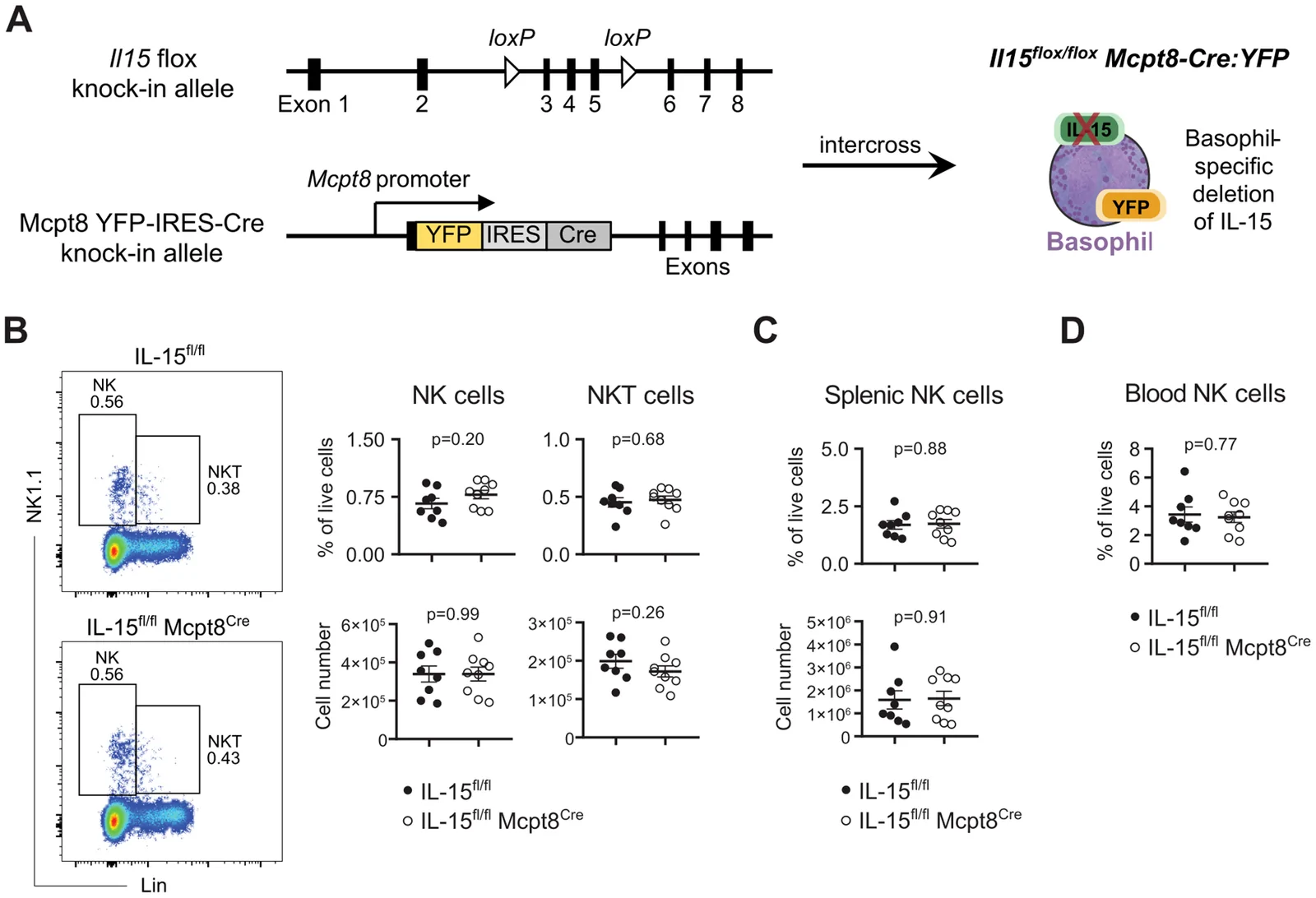
Conditional knockout of IL-15 in basophils has no effect on the generation and survival of NK cells. **(A)** Schematic of the basophil-specific IL-15 conditional knockout mouse model. Mcpt8-Cre: YFP mice, in which Cre recombinase and YFP are expressed under control of the basophil-specific Mcpt8 promoter, were crossed to a homozygous *Il15* floxed background. In the resulting Mcpt8-Cre *Il15^fl/fl^* offspring, Cre-mediated recombination deletes *Il15* in basophils. YFP expression marks basophils that underwent Cre-mediated recombination. **(B)** Representative flow cytometry plots of NK and NKT cells in the bone marrow of *Il15^fl/fl^* and *Il15^fl/fl^ Mcpt8^Cre/wt^* mice. **(C)** Relative and absolute abundance of NK and NKT cells in the bone marrow of *Il15^fl/fl^* control mice and *Il15^fl/fl^ Mcpt8^Cre/wt^* mice. **(D)** Relative and absolute abundance of NK cells in the spleen of *Il15^fl/fl^* and *Il15^fl/fl^ Mcpt8^Cre/wt^* mice. **(E)** Relative abundance of NK cells in the peripheral blood of *Il15^fl/fl^* and *Il15^fl/fl^ Mcpt8^Cre/wt^* mice. All dot plots show mean ± SEM. Numbers above dot plots indicate *P* values from unpaired two-tailed Student’s *t* test.

In order to investigate the functional relevance of basophil-derived IL-15 *in vivo*, we next generated a basophil-specific IL-15 conditional knockout mouse model. For this purpose, mice carrying a heterozygous Cre recombinase knock-in at the endogenous *Mcpt8* locus (Mcpt8-Cre: YFP strain) were crossed with *Il15* floxed mice to a homozygous *Il15^fl/fl^* background. In this model, heterozygous Cre expression is restricted to basophils, allowing targeted ablation of IL-15 production (**Figure 3A**). Flow cytometric analysis of bone marrow cells in *Il15^fl/fl^ Mcpt8^Cre/wt^* mice revealed that Mcpt8-YFP-expressing cells display a basophil phenotype (**Supplementary Figure 3A**) and no Mcpt8-YFP signal was present in other bone marrow cell types, including lymphocytes, monocytes, neutrophils and monocyte progenitors (**Supplementary Figure 3B**). Furthermore, quantification of *Il15* mRNA of sort-purified basophils, Ly6C^hi^ monocytes and neutrophils from the bone marrow of *Il15^fl/fl^* and *Il15^fl/fl^ Mcpt8^Cre/wt^* mice demonstrated specific deletion of *Il15* in basophils but not monocytes or neutrophils (**Supplementary Figures 3C–H**). In contrast to bone marrow neutrophils, bone marrow basophils expressed *Il15ra*, although to a lower level than bone marrow monocytes (**Supplementary Figure 3I**).

Further characterization of the basophil-specific IL-15 knockout mouse model revealed that the frequency of bone marrow basophils and their expression of *Il4*, *Il3ra*, *Mcl1* and *Mki67* was not altered between *Il15^fl/fl^*/*Il15^fl/wt^ Mcpt8^Cre/wt^* and *Il15^fl/fl^ Mcpt8^Cre/wt^* mice (**Supplementary Figures 4A–C**).

Our previous analyses demonstrated that basophils are relatively abundant in the bone marrow, are spatially dispersed throughout the marrow cavity, and represent a cell type with the highest IL-15 expression level among hematopoietic cells. We therefore next asked whether basophil-derived IL-15 contributes to the regulation of IL-15-dependent immune cell populations within the bone marrow microenvironment. First, we assessed whether basophil-derived IL-15 contributes to the maintenance of NK and NKT cells, which are known to critically depend on IL-15 both for their development and homeostasis. Flow cytometric analysis revealed that NK and NKT cell frequencies and absolute numbers in the bone marrow were comparable between *Il15^fl/fl^* and *Il15^fl/fl^ Mcpt8^Cre/wt^* mice (**Figures 3B, C**). Similarly, no differences were observed in the abundance of NK cells in the spleen (**Figure 3D**) or peripheral blood (**Figure 3E**). These findings indicate that basophil-derived IL-15 is dispensable for NK and NKT cell homeostasis under steady-state conditions.

### 3.4 Basophil-derived IL-15 contributes to the maintenance of memory CD8^+^ T cells in the bone marrow

**Figure 4:**
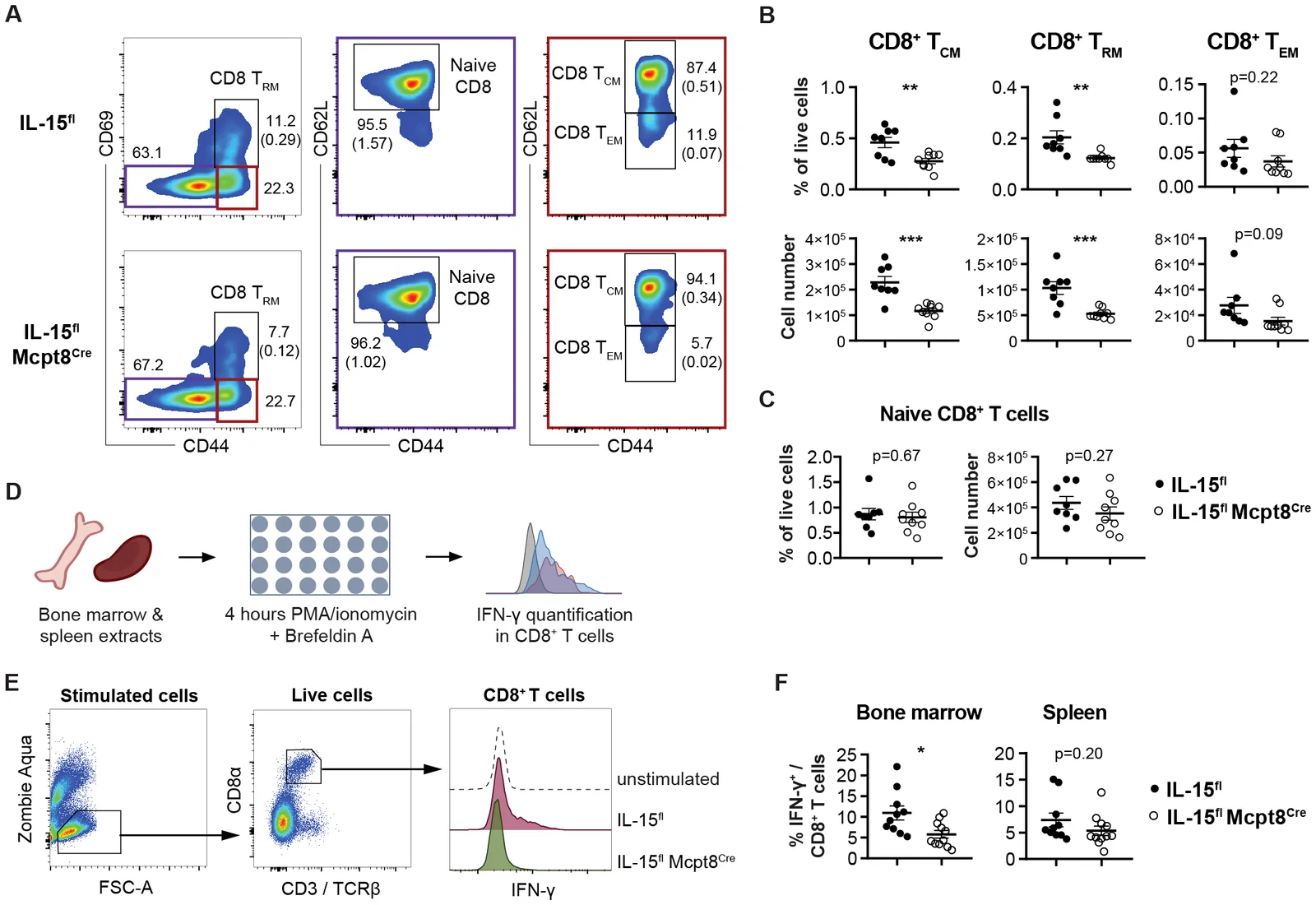
Basophil-specific IL-15 deletion impairs the maintenance of memory CD8^+^ T cell subsets in the bone marrow. **(A)** Representative flow cytometric gating strategy showing the identification of naïve CD8^+^ T cells, CD69^+^ tissue-resident memory (CD8^+^ T_RM_), central-memory (CD8^+^ T_CM_), and effector-memory (CD8^+^ T_EM_) CD8^+^ T cells in the bone marrow in *Il15^fl/fl^* and *Il15^fl/fl^ Mcpt8^Cre/wt^* littermates. **(B)** Relative and absolute abundance of memory CD8^+^ T cell subsets in the bone marrow of *Il15^fl/fl^* and *Il15^fl/fl^ Mcpt8^Cre/wt^* mice. **(C)** Relative and absolute abundance of naïve CD8^+^ T cells in the bone marrow of *Il15^fl/fl^* and *Il15^fl/fl^ Mcpt8^Cre/wt^* littermates. **(D)** Workflow showing the functional analysis of CD8^+^ T cells following *ex vivo* stimulation. Bone marrow and splenic cells were stimulated with PMA/ionomycin in the presence of brefeldin A, followed by intracellular staining of IFN-γ in CD8^+^ T cells. **(E)** Representative histograms for the quantification of IFN-γ-expressing CD8^+^ T cells from *Il15^fl/fl^* and *Il15^fl/fl^ Mcpt8^Cre/wt^* mice. **(F)** Relative and absolute abundance of IFN-γ-expressing CD8^+^ T cells in the bone marrow and spleen from *Il15^fl/fl^* and *Il15^fl/fl^ Mcpt8^Cre/wt^* mice following ex vivo stimulation as indicated in **(D)**. All dot plots show mean ± SEM. Asterisks indicate statistically significant differences and numbers indicate *P* values from unpaired two-tailed Student’s *t* test. *P < 0.05; **P < 0.01, ***P < 0.001.

In addition to NK and NKT cells, IL-15 plays a key role in the maintenance of memory CD8^+^ T cells. Previous studies have demonstrated that global IL-15 deficiency results in a pronounced reduction of memory CD8^+^ T cells (12, 32). We therefore examined whether basophil-derived IL-15 influences memory CD8^+^ T cell compartments in the bone marrow. Using flow cytometry, we first quantified naïve CD8^+^ T cells (CD44^-^CD62L^+^CD69^-^), CD69^+^ memory CD8^+^ T cells that have been proposed to represent bone marrow-resident memory CD8^+^ T cells (33) and frequently express CXCR3 (CD8^+^ T_RM_, CD44^+^CD69^+^; **Supplementary Figure 5A**), central-memory CD8^+^ T cells (CD8^+^ T_CM_, CD44^+^CD62L^+^CD69^-^), and effector-memory CD8^+^ T cells (CD8^+^ T_EM_, CD44^+^CD62L^-^CD69^-^) in the bone marrow of *Il15^fl/fl^* and *Il15^fl/fl^ Mcpt8^Cre/wt^* mice (**Figure 4A**). Strikingly, basophil-specific deletion of IL-15 resulted in a significant 40% reduction of both CD8^+^ T_CM_ and CD69^+^ CD8^+^ T_RM_ cell populations in the bone marrow (**Figure 4B**). In line with this, the frequency of CXCR3^+^ CD8^+^ T cells, which represents a population of cells with Th1 polarization and immediate effector function (34, 35), was also significantly reduced in *Il15^fl/fl^ Mcpt8^Cre/wt^* compared with wild-type littermates (**Supplementary Figure 5B**). The frequency of naïve CD8^+^ T cells was unchanged between wild-type and *Il15^fl/fl^ Mcpt8^Cre/wt^* mice (**Figure 4C**), consistent their independence from IL-15 for survival. To exclude potential Cre-related effects independent of IL-15 deletion, we also analyzed mice heterozygous for the floxed *Il15* allele (*Il15^fl/wt^ Mcpt8^Cre/wt^*). In these animals, CD8^+^ T_CM_ numbers were comparable to control mice, indicating that the observed reduction in memory CD8^+^ T cells depends on IL-15 deletion rather than Cre expression itself (**Supplementary Figure 5C**). As an additional control, we assessed the frequency of central-memory CD4^+^ T cells (CD4^+^ T_CM_, CD44^+^CD62L^+^CD69^-^) in the bone marrow, because they are known to not depend on IL-15 for their survival (32). In contrast to CD8^+^ T_CM_ cells, CD4^+^ T_CM_ cell abundance in the bone marrow was not altered in basophil-specific IL-15 knockout mice (**Supplementary Figure 5D**). In the spleen and peripheral blood, CD8^+^ T_CM_ frequencies were not significantly reduced in *Il15^fl/fl^ Mcpt8^Cre/wt^* mice (**Supplementary Figures 5E, F**), suggesting that the effect of basophil-derived IL-15 on memory CD8^+^ T cell maintenance is most pronounced within the bone marrow. Basophils from bone marrow, spleen, and peripheral blood displayed high frequencies of IL-15-GFP^+^ cells, confirming that they are a robust IL-15-producing population across multiple compartments. However, basophils in the bone marrow had a slightly higher proportion of IL-15-GFP^+^ cells compared to basophils in the spleen (**Supplementary Figure 5G**). Short-term depletion of basophils using an anti-FcϵR1α antibody (MAR-1) was efficient in depleting bone marrow basophils but did not affect the frequency of CD8^+^ T_CM_, CD69^+^ CD8^+^ T_RM_ and NK cells in the bone marrow (**Supplementary Figures 6A–D**). These findings suggest that established (virtual) memory CD8^+^ T cells in the bone marrow of adult mice are not dependent on IL-15 from basophils in the short term.

To determine whether basophil-derived IL-15 also affects the functional capacity of CD8^+^ T cells, we assessed cytokine production following short-term *ex vivo* stimulation. Bone marrow and splenic cells were stimulated with PMA and ionomycin in the presence of brefeldin A, followed by intracellular staining for interferon-γ (IFN-γ) (**Figure 4D**). Notably, CD8^+^ T cells from the bone marrow of *Il15^fl/fl^ Mcpt8^Cre/wt^* mice exhibited a significantly reduced frequency of IFN-γ-producing CD8^+^ T cells compared with controls (**Figures 4E, F**). This decrease was due to the lower frequency of memory CD8^+^ T cells, since the frequency of IFN-γ-producing cells per CD44^+^ memory CD8^+^ T cell was similar in the bone marrow between *Il15^fl/fl^* and *Il15^fl/fl^ Mcpt8^Cre/wt^* mice (**Supplementary Figures 7A, B**). While splenic CD8^+^ T cells also had, on average, a lower frequency of IFN-γ between genotypes, the difference was not statistically significant (**Figures 4E, F**). Together, these data demonstrate that IL-15 produced by basophils contributes to the homeostatic maintenance of IFN-γ-expressing CD8^+^ T cells within the bone marrow microenvironment.

Finally, we assessed *Il15* expression in basophils upon *in vitro* stimulation and during allergic skin inflammation *in vivo* using published RNA-seq and scRNA-seq datasets. For antigen/IgE stimulation, Ito et al. isolated basophils from the mouse bone marrow, pre-incubated them with 2,4,6-trinitrophenol (TNP)-specific IgE followed by stimulation with TNP-ovalbumin (36). Their data showed that *Il15* mRNA levels were significantly reduced in antigen/IgE-stimulated bone marrow basophils two hours after stimulation (**Supplementary Figure 8A**). Cytokine-mediated stimulation of bone marrow basophils using IL-3 and IL-33 performed by Takahashi et al. (37) also resulted in a reduction of *Il15* mRNA levels in basophils four hours after stimulation (**Supplementary Figure 8B**). Using an *in vivo* model of allergic skin inflammation, the scRNA-seq data generated by Imanishi et al. (38) revealed that basophils and neutrophils exhibited the highest levels of *Il15* mRNA expression among the 18 cell types analyzed (**Supplementary Figures 8C, D**). These data suggest that basophils express high levels of *Il15* mRNA during homeostasis but down-regulate *Il15* mRNA upon stimulation with antigen/IgE or IL-3 + IL-33 *in vitro*.

## 4 Discussion

IL-15 is a pleiotropic cytokine that regulates the development, homeostasis and activation of multiple immune cell subsets. It is essential for the development and survival of NK and NKT cells, and supports the long-term functional maintenance of memory CD8^+^ T cells, intraepithelial lymphocytes and γδ T cells (39, 40). Myeloid cells, particularly monocytes, macrophages and CD8^+^ dendritic cells, have long been considered the primary sources of IL-15 that sustain NK cells and memory CD8^+^ T cells. However, studies using IL-15 reporter mice indicate that a broad range of cell types, including mesenchymal stromal cells and endothelial cells, are capable of producing IL-15 (9–11). Of note, data from IL-15 Emerald GFP knock-in/knock-out reporter mice suggest that hematopoietic populations in addition to monocytes and dendritic cells may express IL-15 (10, 30). Yet, these observations have not been systematically validated or assessed for their functional relevance *in vivo*.

In this study, we therefore reanalyzed scRNA-seq data of bone marrow hematopoietic cells from two published studies (25, 26) and found that basophils exhibited both the highest proportion of IL-15-expressing cells and the highest IL-15 expression level among all hematopoietic cells in the bone marrow. Because scRNA-seq exhibits limited sensitivity for transcripts expressed at low or intermediate levels due to stochastic capture inefficacy and dropout effects (11, 31), we used IL-15-IRES-EGFP reporter mice (11) in combination with flow cytometry to comprehensively assess IL-15 expression among hematopoietic populations in the bone marrow and to validate our scRNA-seq findings. Our analyses revealed that IL-15 was expressed by almost all basophils and by 70% of eosinophils and mast cell precursors, whereas fewer than 40% of cells in other myeloid subsets expressed IL-15. Importantly, basophils exhibited the highest IL-15-EGFP expression level of all hematopoietic cells, and was at least twice as high compared to other granulocyte subsets (eosinophils, neutrophils) and monocytes.

To characterize the distribution of basophils in the bone marrow, we reanalyzed a publicly available spatial transcriptomics dataset. This analysis revealed that basophils are evenly distributed throughout the bone marrow cavity, without evident enrichment in either endosteal or perivascular regions. Consistent with this, our flow cytometric data, together with a published study, indicate that basophils constitute a significant fraction of bone marrow cells, accounting for about 0.5 - 0.8% of total bone marrow cellularity (41). Mature basophils also express CD49b and CD29, which together form the integrin α2β1 complex. Primary ligands of CD49b are type I collagen, laminin and fibronectin. Accordingly, mature basophils were found to be enriched in type I collagen-enriched niches in the bone marrow (42). The spatial organization of basophils within extracellular matrix (ECM)-enriched bone marrow microenvironments may allow them to function as widely distributed regulatory cells, capable of modulating multiple hematopoietic compartments. Notably, basophils have been shown to support plasma cell survival in the bone marrow by secreting IL-4 and IL-6 (7). Basophils therefore function within a coordinated network of bone marrow niche cells, including VCAM-1^+^ Lepr^+^ mesenchymal stromal cells and endothelial cells that produce CXCL12 to retain CXCR4^+^ plasma cells, as well as eosinophils and monocytes that express a proliferation-inducing ligand (APRIL), a key plasma cell survival factor (3, 8, 43, 44). Our spatial analysis did not reveal prominent clustering of basophils and CD8^+^ T cells in specialized, dense niches in the bone marrow. This is consistent with a previous study, which reported scattered IL-15 expression in the bone marrow without prominent clustering between IL-15-expressing cells and NK cells (13). However, the enlarged spatial transcriptomics figure (**Figure 1B**) shows that about 60% of basophils (black arrows) in the bone marrow are in contact with CD3^+^CD8^+^ T cells (T_NK). In contrast to hematopoietic stem cells, basophils and memory CD8^+^ T cells are highly motile, and transient interactions between these cell populations appear to be sufficient to deliver IL-15 signals required for memory CD8^+^ T cell survival. We also showed that bone marrow basophils express detectable levels of *Il15ra* mRNA, although at a lower level than bone marrow monocytes. However, it was demonstrated that low expression of *Il15ra* in bone marrow stromal cells resulted in a significant reduction of memory CD8^+^ T cells in *Il15ra^fl/fl^ Osx-Cre* mice (14), similar to *Il15^fl/fl^ Osx-Cre* mice (11).

Conditional knockout studies in mice have shown that IL-15 expression in macrophages and dendritic cells regulates the survival of memory CD8^+^ T cells and NK cells in the bone marrow (12, 13). In addition, distinct IL-15-expressing stromal cells contribute to the maintenance of memory CD8^+^ T cells, NK cells and NKT cells in the bone marrow (11, 14). In humans, the bone marrow has also been shown to host polyfunctional memory CD4^+^ and CD8^+^ T cells with close contact to IL-15-producing cells (15). Although IL-15 expression has been identified in a variety of hematopoietic cell types, including monocytes, dendritic cells, granulocytes and hematopoietic stem and progenitor cells (30), the functional relevance of IL-15 from these populations has so far only been investigated for monocytes and dendritic cells using conditional knockout mouse models (12, 13). Despite evidence that basophils participate in cytokine-driven crosstalk with T cells and other immune cells (45–47), the role of basophil-derived IL-15 in immune cell regulation under physiological conditions has not yet been investigated. To investigate the functional relevance of IL-15 produced by basophils *in vivo*, we crossed IL-15 flox mice (23) with *Mcpt8-Cre* mice, which have been shown to mediate basophil-specific gene deletion (22). Our results demonstrate that basophil-derived IL-15 supports the maintenance of central-memory and CD69^+^ tissue-resident memory CD8^+^ T cells in the bone marrow, but is dispensable for the homeostasis of bone marrow NK and NKT cells. Cell lineages that do not rely on IL-15 for survival, such as memory CD4^+^ T cells and naïve CD8^+^ T cells, remained unaffected by basophil-specific IL-15 deletion. Together, these findings indicate a cell type-specific specialization of bone marrow niches, in which distinct IL-15 sources selectively support particular lymphocyte populations. This is reminiscent of plasma cells, which are sustained by a heterogeneous network of supporting cells, including mesenchymal stromal cells, monocytes, eosinophils and basophils, each producing unique combinations of critical survival factors, such as IL-6, APRIL and CXCL12 (7, 8, 43, 44). Likewise, IL-15 produced by monocytes supported memory CD8^+^ T cell homeostasis in the bone marrow, whereas IL-15 from CD11c^+^ dendritic cells was indispensable for memory CD8^+^ T cell survival in the bone marrow (12). In addition, IL-15-expressing stromal cell subsets differentially supported IL-15-dependent immune cell lineages in the bone marrow. For instance, IL-15-expressing Osx1^+^ stromal cells maintained CD69^+^ tissue-resident memory CD8^+^ T cells and NKT cells but not mature NK cells within the bone marrow (11). Interestingly, basophil-specific IL-15 deletion did not affect memory CD8^+^ T cell frequency in the spleen and blood. This might be due to the lower frequency of basophils in the spleen and blood compared to the bone marrow. Of note, memory CD8^+^ T cells and NK cells in the blood and spleen were found to be primarily regulated by IL-15 produced by endothelial cells, monocytes and dendritic cells (11–14). Another contributing factor may be the unique cellular composition within each microenvironmental niche, which is critical for the maintenance of distinct IL-15-dependent lymphocyte populations. IL-15 may therefore promote the selective expression of integrins and reinforce chemokine receptor programs, thereby coordinating cell adhesion, bone marrow tissue residency and homeostatic proliferation (11, 15, 48).

The reduction in IFN-γ-expressing CD8^+^ T cells was attributable to a shift in the naïve-to-memory CD8^+^ T cell ratio. This interpretation is consistent with the absence of a significant effect on the frequency of IFN-γ-expressing CD8^+^ T cells in the spleen, where basophil frequency is 8-fold lower than in the bone marrow (**Figure 1A**) and where no reduction in memory CD8^+^ T cells was observed in the spleen of *Il15^fl/fl^ Mcpt8^Cre/wt^* mice (**Supplementary Figure 5E**).

In summary, our data identify basophils as a key source of IL-15 in the bone marrow that selectively support memory CD8^+^ T cells under steady-state conditions, thereby uncovering a previously unrecognized role of basophils in supporting homeostatic maintenance of central-memory and CD69^+^ tissue-resident memory CD8^+^ T cells in the bone marrow. This finding further supports the role of basophils as multifunctional, context-dependent regulators of immunity (49, 50), beyond their classical association with allergic inflammation and their contribution to host defense against pathogens.

Limitations of the study: The functional significance of the decrease in bone marrow central-memory and CD69^+^ tissue-resident memory CD8^+^ T cells resulting from basophil-specific IL-15 deficiency, particularly in the context of infections and recall responses, remains to be elucidated.

## Data Availability

Publicly available datasets were analyzed in this study. This data can be found here: Xenium spatial transcriptomics: https://www.10xgenomics.com/datasets (mouse femur bone marrow 2023); RNA-seq: GEO accession GSE272532 and GSE234541; scRNA-seq: GEO accession GSE122465, GSM3466897 and GSE288250 (Tabula Muris, doi:10.1038/s41586-018-0590-4; total bone marrow).
