# Supplementary material for: Basophil-specific deletion of interleukin 15 impairs memory CD8+ T cell homeostasis in the bone marrow
Source: Front Immunol. 2026 Aug 18;17:1842739. doi: 10.3389/fimmu.2026.1842739 (PMC13529516; doi:10.3389/fimmu.2026.1842739)
Supplement: Supplementary file 1 [file DataSheet1.pdf]

Supplementary Material

1.1 Supplementary Figures

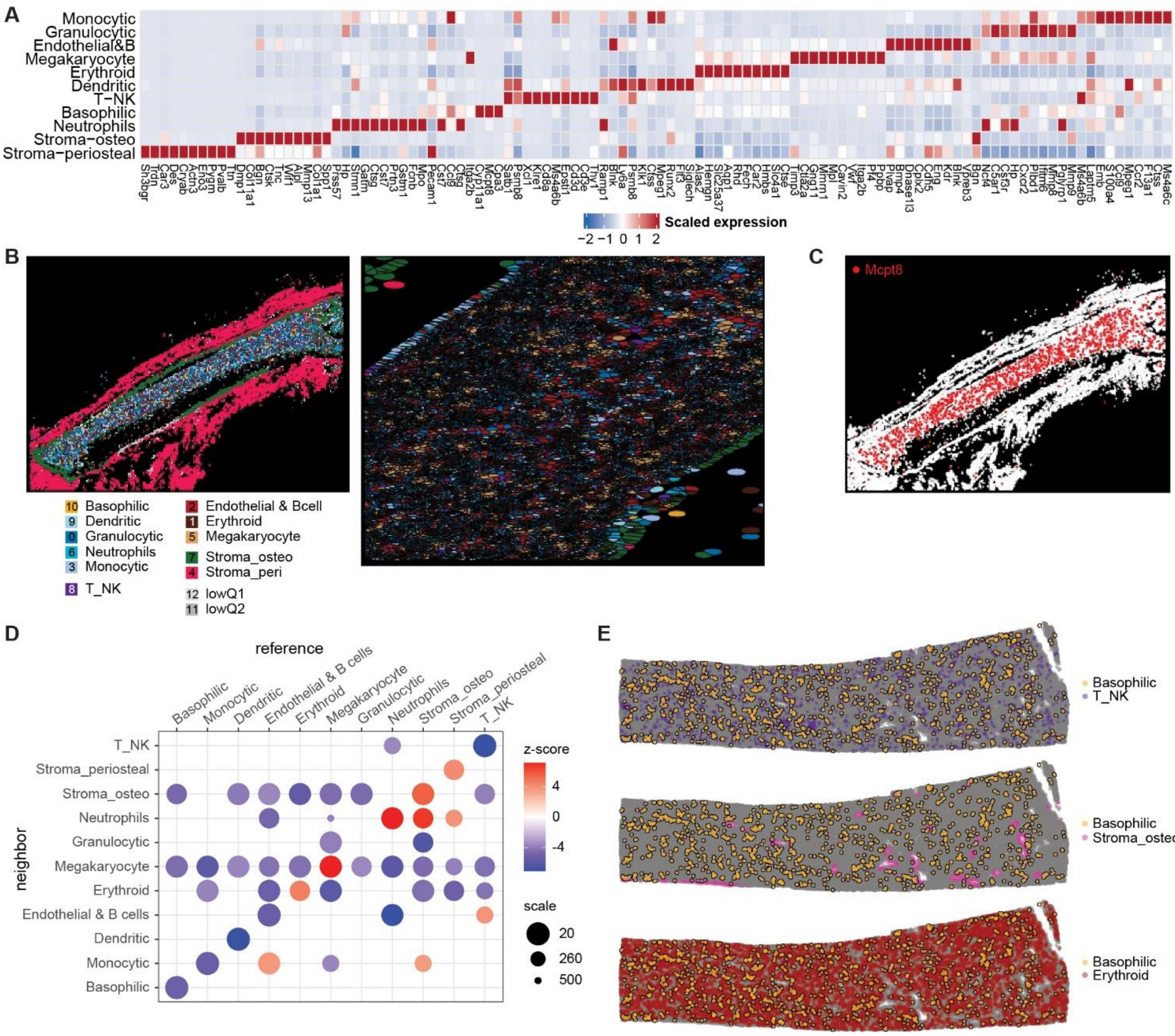

Supplementary Figure 1. Spatial distribution and spatial relationships of basophils in the bone marrow. (A) Heatmap showing scaled expression of curated lineage-defining marker genes across clusters in the murine femur Xenium dataset. **(B)** Spatial distribution of cell clusters in the Xenium dataset. Left: ImageDimPlot showing all annotated cell types across the entire tissue section. Right: Enlarged region highlighting the cellular composition within the bone marrow cavity. **(C)** Spatial feature plot showing expression of the basophil-specific gene *Mcpt8* across the femur section. **(D)** CRAWDAD spatial colocalization analysis summarizing pairwise spatial relationships between the annotated cell types. Dot color represents the z-score of spatial association (positive values indicate enrichment, negative values indicate depletion), and dot size indicates the spatial scale at which the interaction first becomes statistically significant. **(E)** Visualization of spatial relationships between basophils and selected cell types using the vizClusters function. Basophils are shown in relation to T/NK cells, stromal osteogenic cells and erythroid cells within a defined spatial neighborhood excluding the periosteal space.

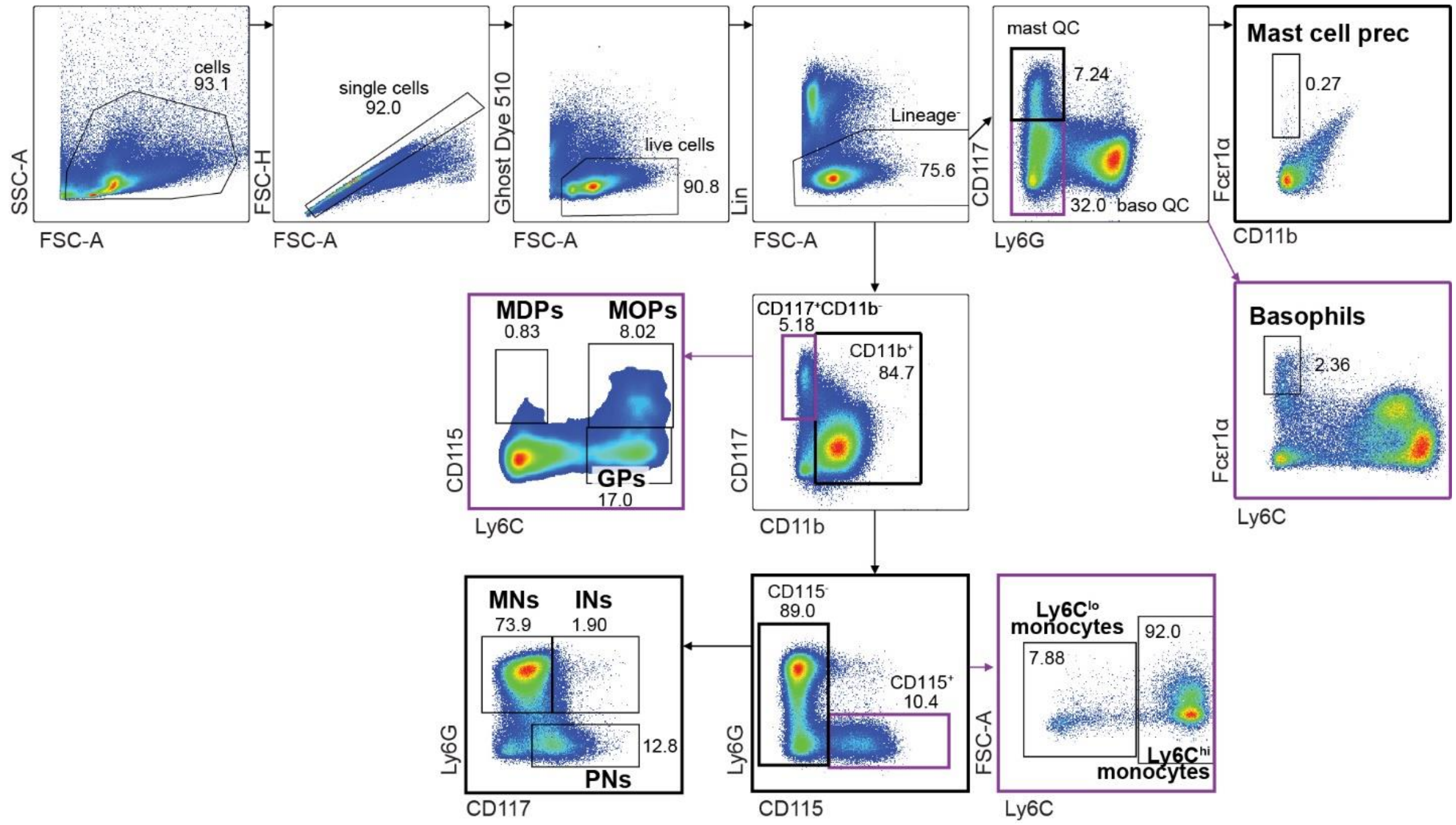

Supplementary Figure 2. Flow cytometric gating strategy for myeloid precursor populations. Representative flow cytometry plots showing the gating strategy used to identify basophils, mast cell precursors, monocyte-dendritic progenitors (MDPs), monocyte progenitors (MOPs), Ly6C^{hi} and Ly6C^{lo} monocytes, granulocyte precursors (GPs), pre-neutrophils (PNs), immature neutrophils (INs) and mature neutrophils (MNs) in the bone marrow of IL-15-GFP reporter mice. The gating strategy has been adopted from Zhang et al. (Zhang et al., 2021).

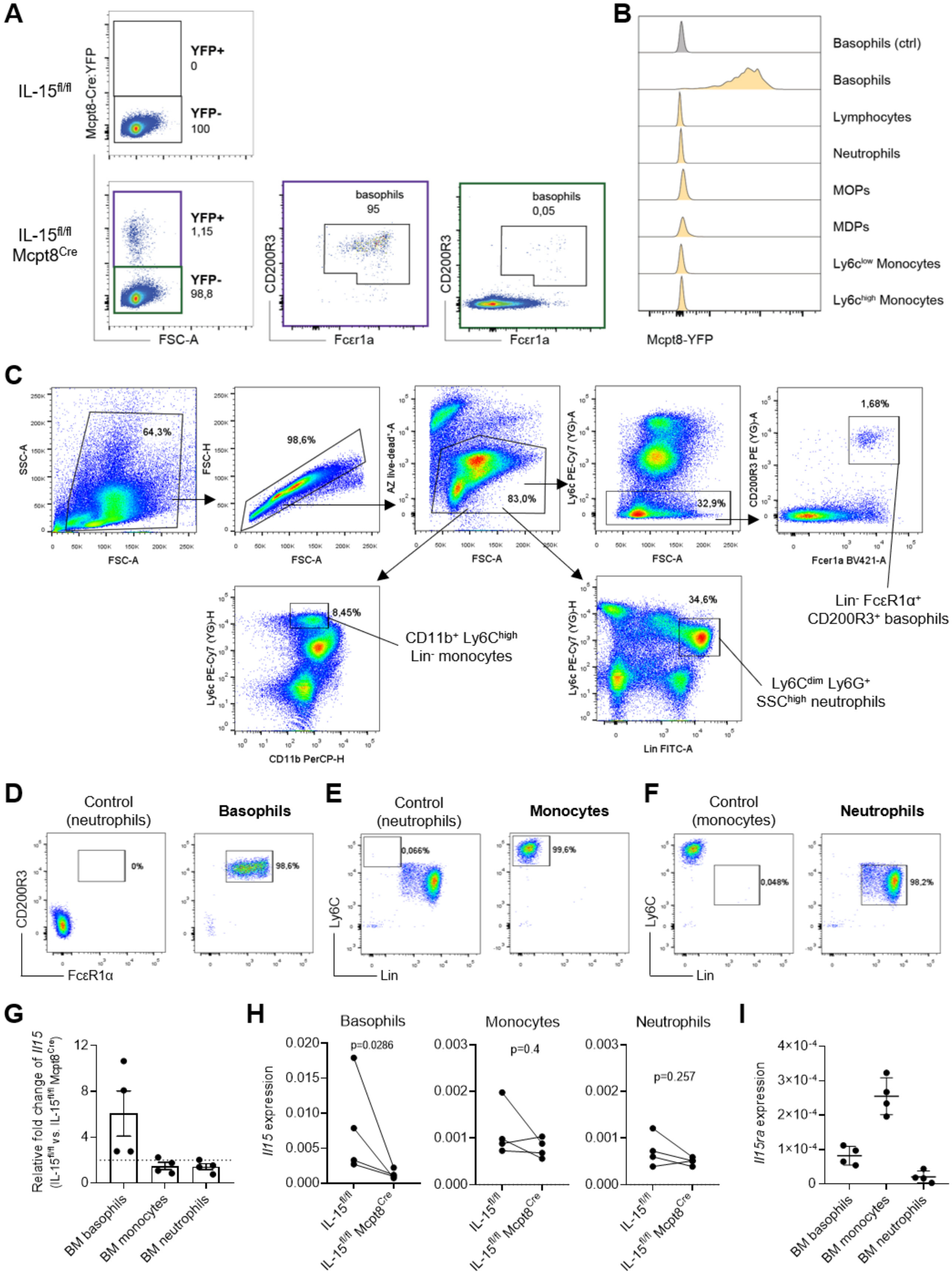

Supplementary Figure 3. Validation of the *Il15$^{fl/fl}$ Mcpt8$^{Cre/wt}$* mouse model.

(**A**) Flow cytometry plots showing the expression of the basophil markers FcεR1α and CD200R among Mcpt8-YFP^{+} and Mcpt8-YFP^{-} cells in the bone marrow. Data are representative of two independent experiments. (**B**) Flow cytometry histograms showing the expression of Mcpt8-YFP in lymphocytes and myeloid cell subsets in the bone marrow. Data are representative of two independent experiments. (**C**) Representative flow cytometry plots showing the gating strategy used to sort-purify Lineage^{-} FcεR1α^{+} CD200R3^{+} basophils, CD11b^{+}Ly6C^{hi} monocytes and Ly6G^{+} Ly6C^{dim} side scatter (SSC)^{high} neutrophils from the bone marrow of *Il15$^{fl/fl}$* and *Il15$^{fl/fl}$ Mcpt8$^{Cre/wt}$* mice for subsequent isolation of RNA. (**D-F**) Representative flow cytometry plots showing the purity of sorted basophils, monocytes and neutrophils from the bone marrow for subsequent isolation of RNA. (**G**) Relative fold change of *Il15* mRNA in sort-purified bone marrow basophils, monocytes and neutrophils (*Il15$^{fl/fl}$* vs. *Il15$^{fl/fl}$ Mcpt8$^{Cre/wt}$*). The dot plot shows mean ± SEM. The dashed line indicates a 2-fold change of *Il15*. (**H**) *Il15* mRNA expression relative to β-Actin in sort-purified basophils, monocytes and neutrophils from the bone marrow of four *Il15$^{fl/fl}$* and four *Il15$^{fl/fl}$ Mcpt8$^{Cre/wt}$* mice. The numbers indicate *P* values from unpaired two-tailed Mann-Whitney U tests. (**I**) *Il15ra* mRNA expression relative to β-Actin in sort-purified basophils, monocytes and neutrophils from the bone marrow of four *Il15$^{fl/fl}$* mice. Abbreviations: MDP, monocyte dendritic cell progenitor; MOP, monocyte progenitor.

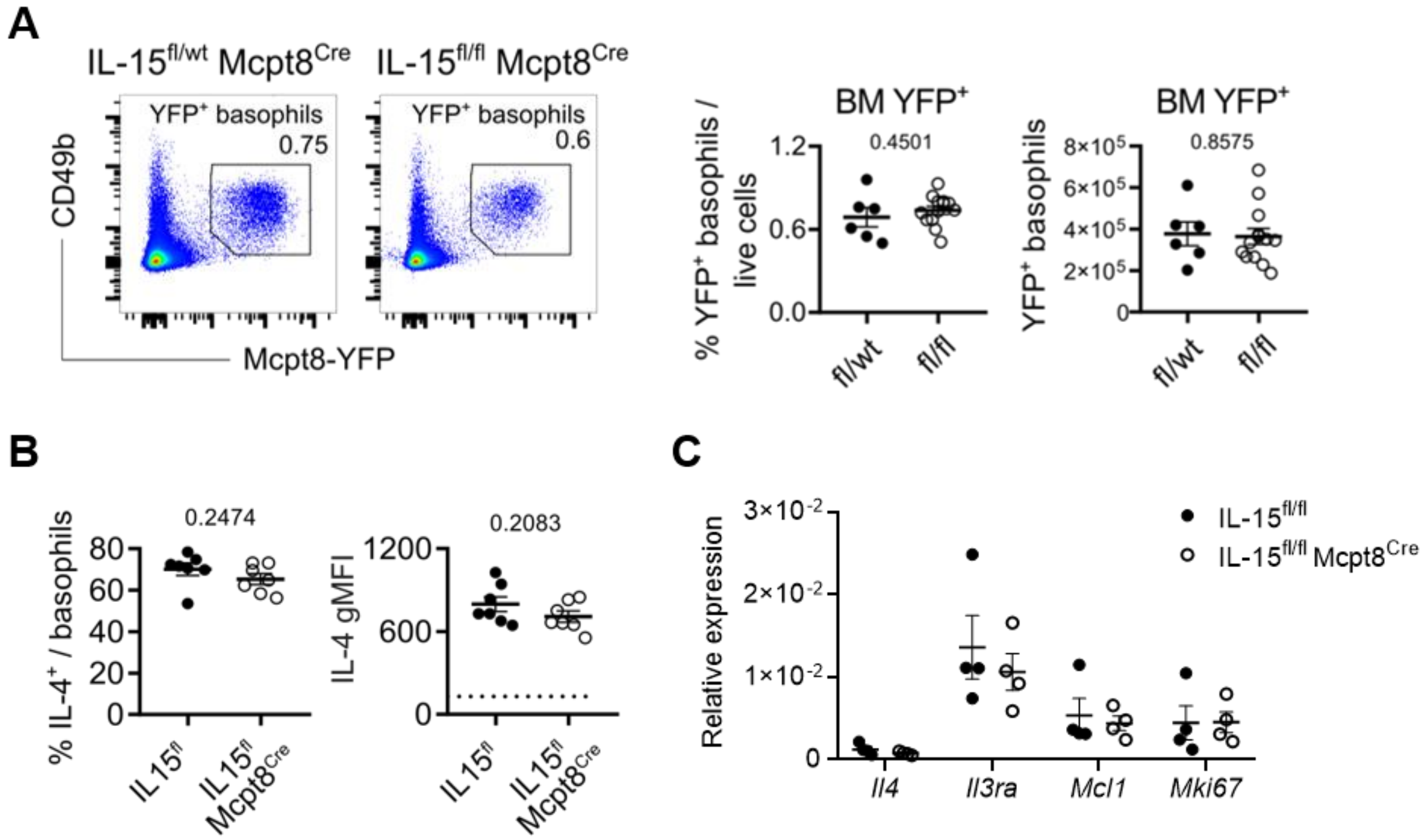

Supplementary Figure 4. Basophil frequency and the expression of key markers in bone marrow basophils of *Il15$^{fl/fl}$*, *Il15$^{fl/wt}$ Mcpt8$^{Cre/wt}$* and *Il15$^{fl/fl}$ Mcpt8$^{Cre/wt}$* mice.

(**A**) Representative flow cytometry plots (left) and dot plot graphs showing the frequency of Mcpt8-YFP^{+} basophils in the bone marrow of *Il15$^{fl/wt}$ Mcpt8$^{Cre/wt}$* and *Il15$^{fl/fl}$ Mcpt8$^{Cre/wt}$* mice. (**B**) Dot plot graphs showing the frequency of IL-4-expressing basophils and the geometric mean fluorescence intensity (gMFI) of IL-4 in basophils from the bone marrow of *Il15$^{fl/fl}$* and *Il15$^{fl/fl}$ Mcpt8$^{Cre/wt}$* mice. (**C**) *Il4*, *Il3ra*, *Mcl1* and *Mki67* mRNA expression relative to β-Actin in sort-purified basophils from the bone marrow of four *Il15$^{fl/fl}$* and four *Il15$^{fl/fl}$ Mcpt8$^{Cre/wt}$* mice. The dot plots show mean ± SEM. The numbers indicate *P* values from unpaired two-tailed Mann-Whitney U tests.

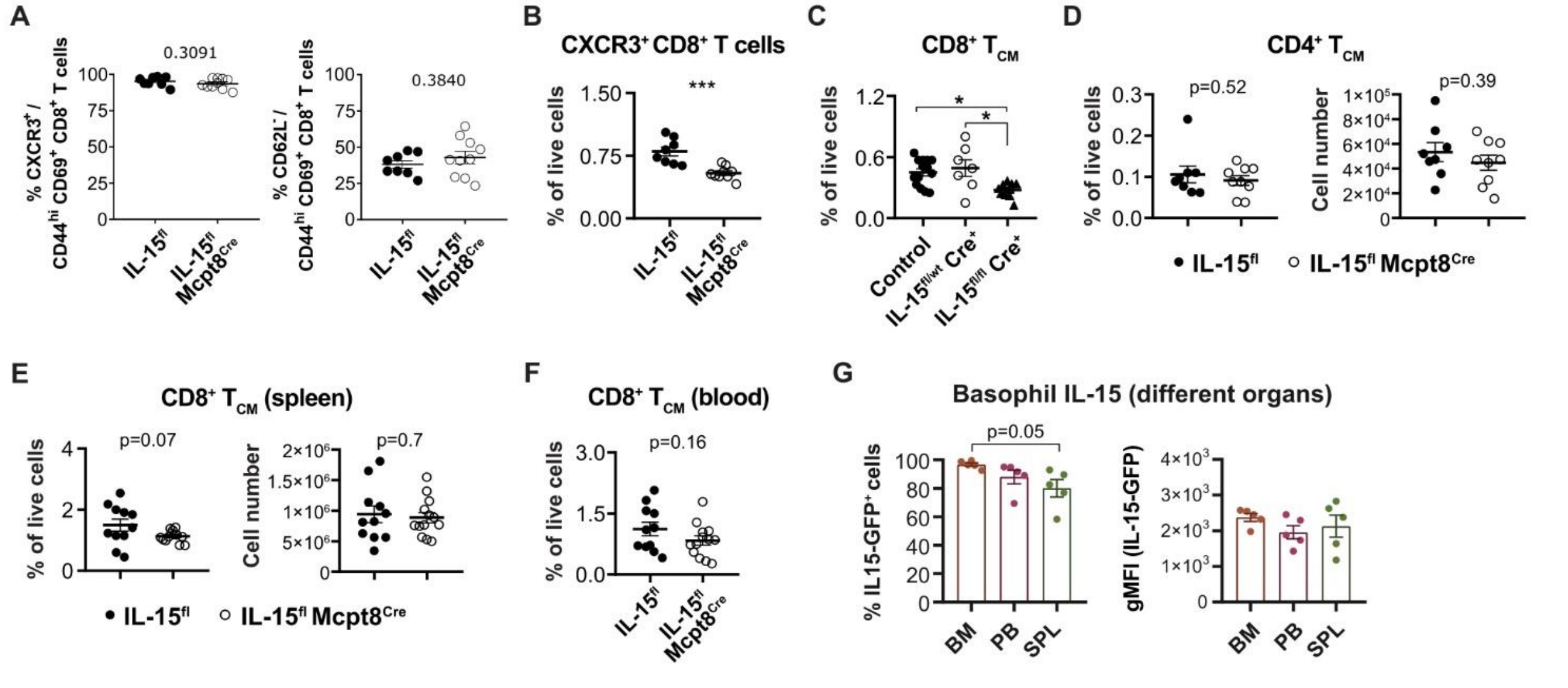

Supplementary Figure 5. Extended characterization of memory T cell phenotypes and basophil IL-15 expression across tissues.

(A) Relative abundance of CXCR3 and CD62L among CD44^{hi} CD69^{+} CD8^{+} T cells in the bone marrow of *Il15$^{fl/fl}$* and *Il15$^{fl/fl}$ Mcpt8$^{Cre/wt}$* littermates. **(B)** Relative abundance of CXCR3^{+} CD8^{+} T cells in the bone marrow of *Il15$^{fl/fl}$* and *Il15$^{fl/fl}$ Mcpt8$^{Cre/wt}$* littermates. **(C)** Relative abundance of central-memory CD8^{+} T cells (CD8^{+} T_{CM}) in control mice (*Il15*$^{fl/wt}$ or *Il15*$^{fl/fl}$), heterozygous basophil IL-15 knockout mice (*Il15$^{fl/wt}$ Mcpt8$^{Cre/wt}$*), and homozygous basophil IL-15 knockout mice (*Il15$^{fl/fl}$ Mcpt8$^{Cre/wt}$*). **(D)** Relative and absolute quantification of the abundance of central-memory CD4^{+} T cells (CD44^{+}CD62L^{+}CD69^{-}) in the bone marrow of *Il15$^{fl/fl}$* and *Il15$^{fl/fl}$ Mcpt8$^{Cre/wt}$* mice. **(E)** Relative and absolute quantification of CD8^{+} T_{CM} in the spleen of *Il15$^{fl/fl}$* and *Il15$^{fl/fl}$ Mcpt8$^{Cre/wt}$* mice. **(F)** Relative quantification of CD8^{+} T_{CM} in the peripheral blood of *Il15$^{fl/fl}$* and *Il15$^{fl/fl}$ Mcpt8$^{Cre/wt}$* mice. **(G)** Bar plots showing IL-15-GFP expression in basophils from bone marrow, spleen, and peripheral blood. Left: percentage of IL-15-GFP positive basophils. Right: geometric mean fluorescence intensity (gMFI) of IL-15-GFP in basophils. All dot plots show mean ± SEM. Asterisks indicate statistically significant differences (* $P < 0.05$, *** $P < 0.001$) and numbers indicate *P* values from unpaired Student's *t* test. One-way ANOVA and Tukey's post hoc test was used to calculate differences for panels B and F.

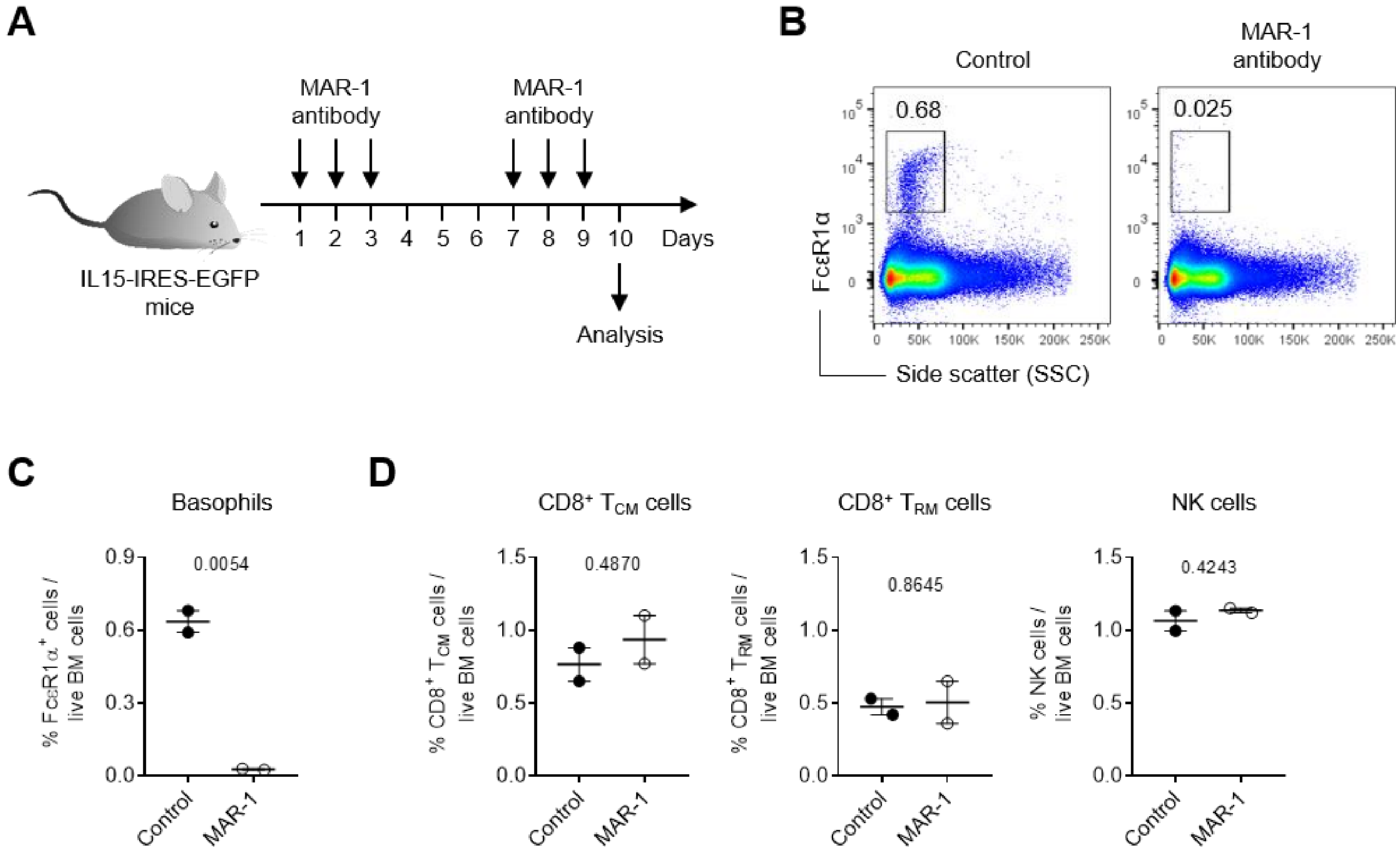

Supplementary Figure 6. Short-term depletion of basophils using MAR-1 monoclonal antibody.
(**A**) Experimental workflow for the short-term depletion of basophils using a MAR-1 monoclonal antibody. (**B**) Representative flow cytometry plots showing the frequency of FcεR1α^{+} SSC^{low} basophils in the bone marrow of a PBS-treated and MAR-1 antibody treated mouse at day 10. (**C**) Dot plot graph showing the frequency of FcεR1α^{+} SSC^{low} basophils in the bone marrow of PBS-treated and MAR-1 antibody treated mice at day 10. (**D**) Dot plot graphs showing the frequency of CD8^{+} T$_{CM}$, CD8^{+} T$_{RM}$ and NK cells (CD3^{-}NK1.1^{+}CD127^{-}CD49b^{+}) in the bone marrow of PBS-treated and MAR-1 antibody treated mice at day 10. The dot plots show mean ± SEM. The numbers indicate *P* values from unpaired two-tailed Student's *t* tests.

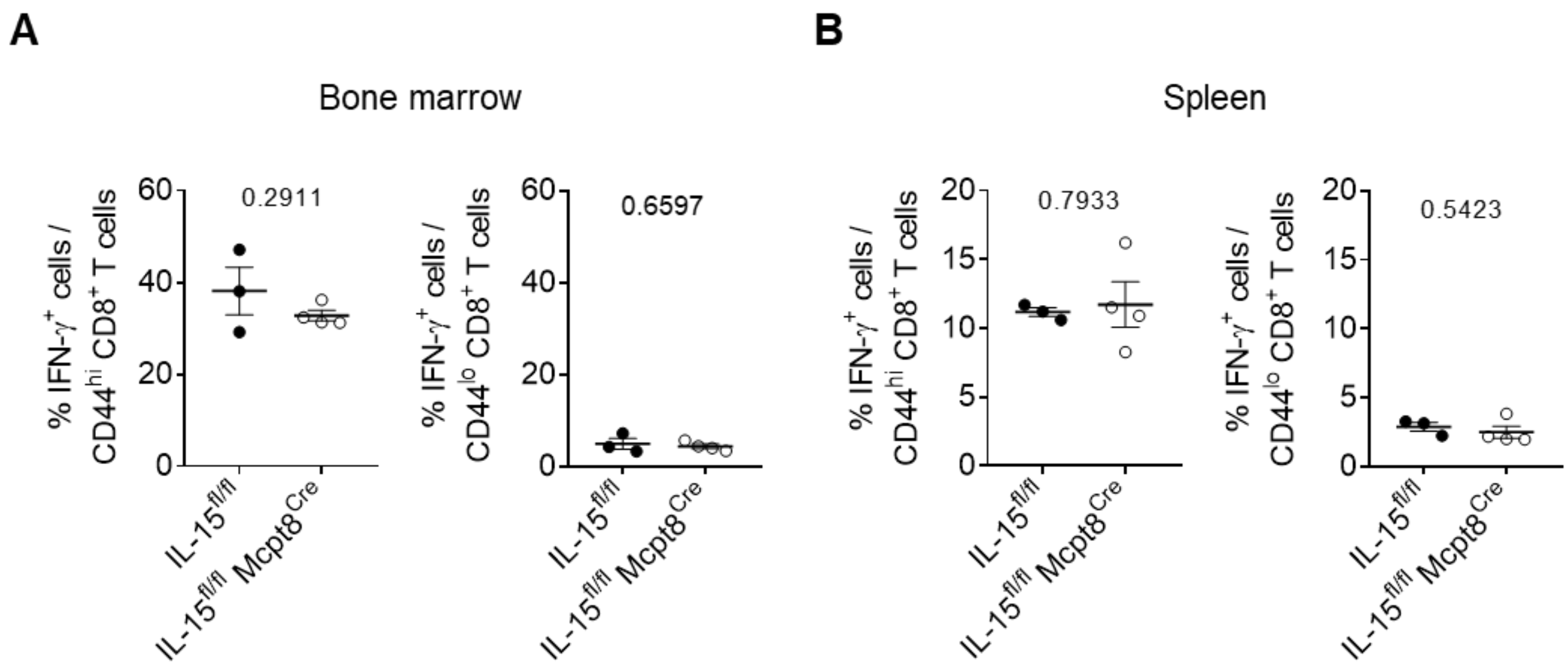

Supplementary Figure 7. Relative abundance of IFN-γ^{+} cells among naïve and memory CD8^{+} T cells. (**A**) Dot plot graphs showing the frequency of IFN-γ^{+} cells among CD44^{hi} memory and CD44^{lo} naïve CD8^{+} T cells in the bone marrow. (**B**) Dot plot graphs showing the frequency of IFN-γ^{+} cells among CD44^{hi} memory and CD44^{lo} naïve CD8^{+} T cells in the spleen. The dot plots show mean ± SEM. The numbers indicate *P* values from unpaired two-tailed Student's *t* tests.

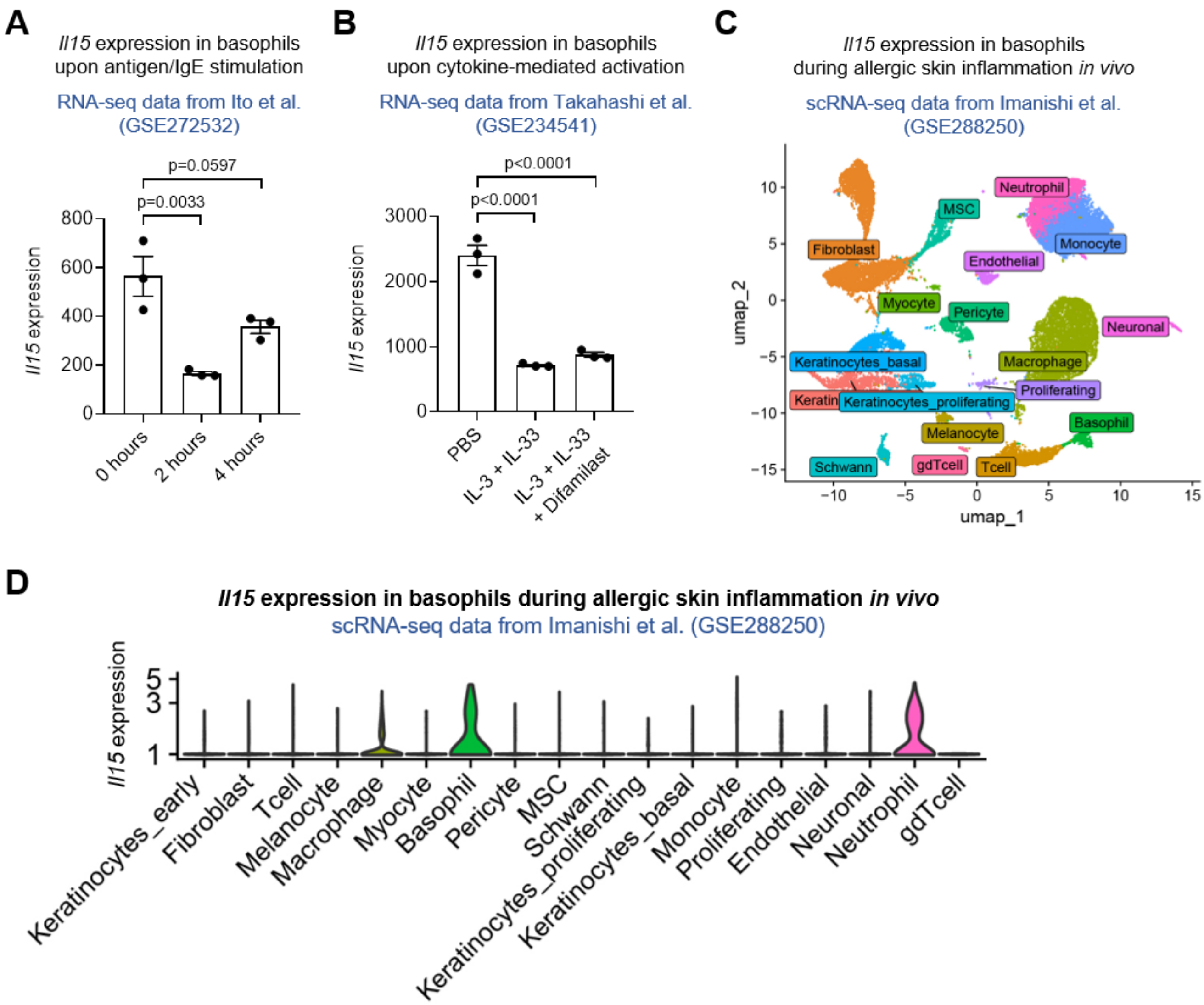

Supplementary Figure 8. *Il15* expression in basophils upon stimulation in vitro and during allergic skin inflammation in vivo.

(**A**) Dot plot graph showing *Il15* mRNA expression in mouse bone marrow-derived basophils pre-incubated with TNP-specific IgE followed by stimulation with TNP-OVA on culture day 6. The RNA-seq dataset was published by Ito et al. (GSE272532) (Ito et al., 2025). (**B**) Dot plot graph showing *Il15* mRNA expression in mouse bone marrow-derived basophils treated with IL-3 + IL-33 for 4 hours. The RNA-seq dataset was published by Takahashi et al. (GSE234541) (Takahashi et al., 2024). (**C**) UMAP plot of immune and stroma cell subsets from a murine type 2 skin inflammation model. The scRNA-seq dataset was published by Imanishi et al. (GSE288250) (Imanishi et al., 2025). (**D**) Violin plots showing *Il15* expression in immune and stroma cell subsets in a murine type 2 skin inflammation model using the scRNA-seq dataset from (C).

References

Imanishi, I., Gill, R., Wilder, A., Restrepo, P., Nair, A., Cho, I., Krueger, J. G., Guttman-Yassky, E., Kim, B. S., and Ji, A. L. (2025). A basophil-fibroblast pro-inflammatory axis fuels type 2 skin inflammation. Cell reports *44*, 116114.

Ito, J., Miyake, K., Chiba, T., Takahashi, K., Uchida, Y., Blackshear, P. J., Asahara, H., and Karasuyama, H. (2025). Tristetraprolin-mediated mRNA destabilization regulates basophil inflammatory responses. Allergol Int *74*, 263-273.

Takahashi, K., Miyake, K., Ito, J., Shimamura, H., Suenaga, T., Karasuyama, H., and Ohashi, K. (2024). Topical Application of a PDE4 Inhibitor Ameliorates Atopic Dermatitis through Inhibition of Basophil IL-4 Production. J Invest Dermatol *144*, 1048-1057 e1048.

Zhang, J., Wu, Q., Johnson, C. B., Pham, G., Kinder, J. M., Olsson, A., Slaughter, A., May, M., Weinhaus, B., D'Alessandro, A., *et al.* (2021). In situ mapping identifies distinct vascular niches for myelopoiesis. Nature *590*, 457-462.
